# A Qualitative Study on the Knowledge, Attitude, and Practices Regarding Schistosomiasis Prevention Among Primary School Children in the Riverine Communities of Osun State, Nigeria

**DOI:** 10.7759/cureus.81209

**Published:** 2025-03-25

**Authors:** Sunday C Adeyemo, Sunday Olarewaju, Eniola D Olabode, Ayodele R Ajayi, Adeola D Aderinwale, Kehinde Awodele, Akintade J Odunlami, Oluwafunmilayo Fasanu

**Affiliations:** 1 Health and Biomedical Sciences, Institut Superieur de Sante, Niamey, NER; 2 Community Medicine, Osun State University, Osogbo, NGA; 3 Psychiatry, Afe Babalola University Teaching Hospital, Ado-Ekiti, NGA; 4 Public Health, Adeleke University, Ede, NGA

**Keywords:** children, knowledge, primary school, river-rine, schistosomiasis

## Abstract

Background

Despite the implementation of severe rounds of preventive chemotherapy, schistosomiasis is a disease of concern affecting millions of people, especially in underdeveloped and developing countries including Nigeria. This study aimed to use a qualitative method to have an in-depth understanding of the gaps in school children's knowledge, attitude, and practices regarding schistosomiasis.

Methods

This study employed a qualitative method to obtain information from schoolchildren in riverine villages in Osun State, Nigeria. The study was conducted among 138 children in Primary 4-6 across 12 schools selected using the purposive sampling technique. A total of 24 focus group discussions were conducted by public health professionals and doctors. The recorded data was transcribed and analyzed using ATLAS.ti software (ATLAS.ti Scientific Software Development GmbH, Berlin, Germany).

Results

A few respondents (27.5%) accurately identified the symptoms, risk factors, and preventive measures of schistosomiasis. A sizable portion (75.9%) expressed readiness to seek care early from professionals within the hospital settings, undergo medical tests to confirm the diagnosis, and adhere to prescribed drugs and other preventive measures appropriately as a primary preventive measure to ensure clearance of organism. A significant portion (78.8%) of the participants indicated the use of pit latrines as the most common method of sewage disposal. Outside the house, the most commonly mentioned is defecating in the bush, reflecting open defecation practices.

Conclusions

This study highlights the gaps in knowledge, attitude, and practices regarding schistosomiasis among children in the riverine communities of Osun State. To reduce the disease, we need targeted health education, better sanitation, and easier access to healthcare. These steps can help protect children and control the disease in the long term.

## Introduction

Neglected tropical diseases (NTDs), which are infectious diseases of poverty, affect nearly one billion people worldwide, particularly in underdeveloped and developing nations where the most vulnerable people live [1]. Schistosomiasis remains a significant public health concern, with about 251.4 million people needing preventive therapy in 2021 [2]. Schistosomiasis has significant negative consequences on the economy and health; people infected with schistosomiasis may experience difficulties at school and work. The disease causes greater morbidity than mortality [1]. The current estimate of deaths from schistosomiasis according to the World Health Organization is 11,792 per year worldwide [2].

Despite the implementation of several rounds of preventive chemotherapy, schistosomiasis remains a disease of concern, affecting millions of people, especially in underdeveloped and developing countries, including Nigeria. Osun State in southwestern Nigeria was classified as hyper-endemic, ranked sixth among 36 states, including the Federal Capital Territory (FCT), in a 50-year review study on the burden and pattern of distribution across different states [3].

Meeting Sustainable Development Goals (SDG) for schistosomiasis control among neglected tropical diseases will require assessing the burden and distribution of infection among school children, identifying barriers hindering the elimination of infection and re-infection, and compliance with healthy behaviors using qualitative techniques among vulnerable groups such as school children in order to develop appropriate context-specific solutions. Hence, this study aimed to use a qualitative method to assess gaps in school children's knowledge, attitude, and practices regarding schistosomiasis in the riverine villages of Osun State. This will serve as the baseline information for future intervention studies.

## Materials and methods

This was a cross-sectional study that employed a qualitative method (Focus Group Discussion) to obtain information from schoolchildren of the riverine villages in Osun State, Nigeria. The study was conducted among 138 children in Primary 4-6 across the different schools. The number of students to be selected in Primary 4, 5, and 6 was determined by proportional allocation. Pupils were selected from public schools while private school pupils were excluded because Nigeria is a classed society where children from private schools are assumed to have better access to resources compared to children in public schools. Pupils in classes below primary 4 were also excluded. The participants were selected from 12 public schools using the purposive sampling technique, which was based on their level of endemicity. A total of 24 focus group discussions (FDGs) using semi-structured questions were done (two per school, male and female respondents separated) between Monday, March 4, 2024 and Friday, March 29, 2024. The FDGs were conducted by public health professionals and doctors and were recorded after written consent was obtained from the parents of the participants and the participants gave verbal consent. The recorded data was transcribed and analyzed using the ATLAS.ti software (ATLAS.ti Scientific Software Development GmbH, Berlin, Germany). Grounded theory was applied during content analysis.

## Results

Socio-demographic characteristics of the participants

The majority of the participants were more than 10 years old (108, 78.3%), Muslims (81, 58.7%), and in Primary 6 (80, 58.7%) (Table 1).

**Table 1:** Socio-demographic Characteristics of the Participants Pry: Primary

Variable	Frequency	Percentages
Age in years		
7-9	30	21.7
> 10	108	78.3
Gender		
Female	69	50
Male	69	50
Class		
Pry 4	23	16.7
Pry 5	35	25.3
Pry 6	80	58
Religion		
Muslim	81	58.7
Christianity	57	41.3

Knowledge regarding schistosomiasis

Table 2 reveals the knowledge of respondents about the mode of transmission, signs and symptoms, and risk factors concerning schistosomiasis. Respondents have divergent opinions, myths, and misconceptions regarding the mode of transmission, signs and symptoms, and risk factors concerning schistosomiasis. A few (27.5%) respondents accurately identified schistosomiasis as a disease that causes discomfort during urination, can also lead to a bloated stomach, and is linked to water-related activities such as bathing and swimming in an infected river.

**Table 2:** Knowledge About Schistosomiasis

Knowledge regarding schistosomiasis	Quotes from the focused group discussions
Signs and symptoms of schistosomiasis	“A sickness that causes discomfort while urinating.”
	“They have bloated stomach.”
Causes and transmission of schistosomiasis	"It occurs if a person goes to bathe in a river that is not safe."
	"Bathing in the river."
	"When bathing/swimming in the river and an insect in the water clings to the person, the person can have schistosomiasis."
	"Bathing in the stream can cause skin infection."
	"Disease can be transferred while playing with dog, leaving food uncovered."
	"... dog barking at someone."
	"... it is caused by a parasite like mosquitoes."
	"... playing with dog."
	"... bitten by dog, playing too much with dog."
Insanitary practices that are risk factors	"Playing with dirt on dumping ground."
	"Playing with sand."
	"Coming in contact with dirty toilet and dirty toilet water"
	"Insect might enter the body while defecating outside through carelessness" and "Insect may enter the body while defecating outside."
	"Sexual intercourse."
	"Urinating in the bush every time."
Preventive practices towards schistosomiasis (type of drug to be used, accessibility, availability as part of preventive measures)	"Staying far from dog."
	"The person should dig the ground before defecating and cover after defecation."
	"It can be bought at a chemist shop."
	"The infected person should use the prescribed drug like paracetamol."
	"Use of vitamin C."
	‘… Alabuku drug."
	"The drugs are costly and not accessible in our vicinity."
	"It is sold at a high price."

However, there were myths and misconceptions about the mode of transmission and risk factors of the disease. Some (32.6%) respondents mentioned transmission through sexual intercourse and urinating in the bush every time. A few (29%) participants mentioned that the disease could be transmitted while playing with dogs, leaving food uncovered, and dogs barking. Many (67.5%) believed that the disease is transmitted by contact with insects during outdoor defecation, sexual intercourse as well as playing with sand and in dirty toilets.

In terms of preventive practices, a greater proportion of respondents (72.5%) interviewed had little or no knowledge of access to water and toilets for improved hygiene and sanitation practices towards the prevention of schistosomiasis while others had limited understanding of the type of drug, accessibility, availability as part of preventive measures.

Attitude towards schistosomiasis

Table 3 reveals the attitude of participants towards the prevention of schistosomiasis. A sizable portion (85.0%) of participants expressed concerns and highlighted the importance of having knowledge and taking preventive action. This group thinks it's important to be concerned about the illness for a number of reasons, especially in relation to personal health and safety as well as the prevention of further complications, and the physical and emotional disturbances associated with the diseases. However, some respondents (42.0%) believed that illness is not a threat to their existence, and hence they didn't care in their response as a result of low perceived risk.

**Table 3:** Attitude Towards Schistosomiasis Prevention ^*^Bilharzia is another word for schistosomiasis.

Attitude towards schistosomiasis prevention	Quotes from focused group discussion
Importance of having knowledge of schistosomiasis and taking preventive action	It is important to know how to prevent it because it is not easy to be infected with the disease; if a person is infected, the person won’t enjoy himself."
	They must go for treatment to be cured."
	"Yes, it is necessary to be bothered about it."
	“It is important to know how to prevent it because it is not easy to be infected with the disease. If a person is infected, the person won’t enjoy himself.”
	"Because it is not easy to be infected with the disease, if a person is infected, the person won’t enjoy himself."
	"It is not compulsory."
Health-seeking behaviour	"The person infected with this disease should go to the hospital, and go for a medical test."
	"Use of drugs prescribed in the hospital appropriately."
	"The person should follow the prescription so that the disease will not multiply."
	"Go to the hospital."
	"Contact doctor."
	"Going to the hospital."
	"The person should practice cleanliness."
	"Take herbs."
	"Herbal treatment."
	"The infected person should consult those selling herb drugs."
Attitude towards screening test regarding schistosomiasis	“I have not checked for the disease before.”
	“I have checked for the disease before, last year at the school.”
	"I will do the checkup and tests."
	"I'll be willing to check for bilharzia so that the disease can go."*
	"We would love to know more."
	"I want to know more."
	"I don’t want to."
	"Never done it, I don’t want to."
Attitude towards adoption of safe sanitary practices and prevention of contact with water	"It is good to be defecating inside the toilet. It is important."
	“It is important because some people might be playing with their urines, which may cause the disease to be transmitted to others.”
	"A fisherman should be careful so that his boat will not capsize."

Regarding health-seeking behaviour related to schistosomiasis infection, a sizable portion (75.9%) expressed the importance of seeking care early from professionals within the hospital setting, undergoing medical tests to confirm the diagnosis, and adhering to prescribed drugs and other preventive measures. However, the use of herbal remedies was mentioned among some (22.5%) participants.

Concerning attitude towards the screening test for schistosomiasis, the majority (90.0%) of the participants reported that they had never been tested. However, a significant number of participants expressed willingness to be screened for schistosomiasis (75.2%), though some (24.8%) participants expressed unwillingness to be screened for schistosomiasis.

Concerning the adoption of safe sanitary practices and prevention of contact with infected water, many participants (75.5%) recognized that the adoption of safe sanitary practices will reduce the prevalence of schistosomiasis as well as other faecal-oral diseases. 

Practices regarding schistosomiasis control

Table 3 shows the practices regarding the prevention of schistosomiasis. Participants mentioned various types of sewage disposal methods used within their houses such as pit latrines, water closets, and potty. The use of pit latrines was most commonly mentioned. The water closet (flush toilet) was least mentioned. Outside the house, the most common method of sewage disposal mentioned was defecating in the bush. It was also mentioned that pit latrines are primarily used by adults, and in some cases, children are actively prevented from using pit latrines and use the bush as an alternative method.

**Table 4:** Practices Related to Schistosomiasis

Practices regarding Schistosomiasis prevention	Quotes from focused group discussion
Sewage disposal method	“Pit latrine.”
	“Bush.”
	“The pit latrine is used by adults and children are prevented from using it.”
	“The pit latrine is used by adults.”
	“Children should use the bush.”
	“Dunghill are used by children, water closet by adults.”
Common water sources and its usage	“Borehole water is used for washing plates.”
	“Borehole water is used in bathing.”
	“Borehole water for cooking.”
	“Well water is used for washing clothes.”
	“Well water for taking bath and for washing clothes.”
	“Well water is fetched in a clay pot for cooking.”
	“River water for fishing activity.”
	“River water for swimming.”
	“River water for molding blocks.”

Concerning common sources of water, various sources of water were mentioned by the participants, including, rainwater, river water, well water, borehole water, and tap water, with well water being the most common. Well water, according to the participants, serves different purposes - the majority use it for cooking while others mentioned that it is used for bathing and laundry. A few of the participants (28.0%) also mentioned the use of river water for swimming and fishing activities and some reported using it for molding blocks, washing clothes, and also for cooking.

## Discussion

This study gives us detailed information about the knowledge, attitude and practices towards schistosomiasis among primary school children in the riverine areas of Osun State, Nigeria. These details can help create better plans to control the disease in these communities.

The results show that while some pupils knew schistosomiasis is linked to water activities like swimming in infected rivers, many had wrong ideas about its mode of transmission. Some believed it could be transmitted through sexual contact, urinating in the bush, playing with dogs, or leaving food uncovered. Others thought it came from insect bites, dirty toilets, or playing in the sand. Such misconceptions was reported in Zimbabwe among third grade primary school student, where consumption of green mangoes, treading on a witch's foot, jumping over fire, and consuming an excessive amount of salty food were mentioned as part of the causes of schistosomiasis infection [4]. Similarly, misinformation, such as consuming too much sugar, drinking dirty water, drinking water that an infected person has urinated in, playing in murky water, and eating cold and undercooked food, were reported in the study of Masaku et al. conducted in Kenya among community leaders and parents [5]. These misunderstandings show that more health education is needed to help pupils understand how schistosomiasis spreads and how to prevent it [2]. Also, many pupils did not know that medicine is available to treat schistosomiasis. According to Aula et al., lack of awareness about available treatments prevents effective disease control [6].

The children’s attitude about the disease were mixed. Some pupils were worried about schistosomiasis and knew it was important to take preventive actions. They understood that the disease could cause serious health problems. They also knew that seeking medical help early, taking prescribed drugs, and following preventive measures were important. This positive attitude shows they are open to learning and changing their behavior, as Torres-Vitolas et al., also found in similar studies [7]. However, others did not see schistosomiasis as a serious threat, which made them less concerned about prevention. A study by Mujumbusi et al. shows that when people do not think they are at risk, they are less likely to take action to prevent the disease [8].

When it came to treatment, many (75.9%) pupils knew they should see a doctor, but some still preferred herbal remedies. Traditional medicine is common in many communities. Also, some children use herbal remedies instead of modern** **medicine to treat schistosomiasis. While traditional medicine has been shown in recent studies to play some role in the treatment of schistosomiasis [9,10], there is still a need to test the efficacy, safety, and quality of herbs in order to prevent further damage to the body system. Also, most pupils had never been tested for schistosomiasis, but many said they were willing to be screened. This suggests that if schools and health centers provide more screening opportunities, more cases could be detected and treated early.

Some of the pupils' daily habits put them at risk of getting schistosomiasis. Many families used pit latrines, but some children were not allowed to use them and instead defecated in the open. Open defecation is a big problem because it allows the schistosomiasis parasite to spread into water sources [2]. Adults in these communities mostly use pit latrines, but children often defecate in bushes, spreading the disease. Some children also swim or fish in rivers, exposing them to infected water. This is similar to the findings of Adeneye et al. in the rural communities of Ondo State, Nigeria where poor sanitation and unsafe water were associated with an increase in the risk of infection [9]. Water sources were another issue. Most pupils used well water for cooking and drinking, but some also used river water for swimming, fishing, and even cooking. Research by Mbereko et al. shows that contact with infected water is one of the main ways schistosomiasis spreads [11]. This means that pupils who swim or fish in contaminated rivers are at a high risk of infection. Schools and communities should teach students how to use safe water and protect themselves from schistosomiasis.

The limitation of this study is that it may not be generalized due to the influence of socio-economic factors. Purposive sampling used could also lead to some bias in selection.

## Conclusions

The findings revealed myths and misconceptions concerning the causes and modes of transmission of schistosomiasis. The majority of the pupils had a good attitude towards schistosomiasis, especially in seeking medical assistance, and were willing to undergo screening, though some of the participants preferred herbal remedies and some were not willing to undergo screening. Poor sanitary practices were also mentioned.

This study highlights the gaps in knowledge, attitude, and practices regarding schistosomiasis among children in the riverine communities of Osun State. To reduce the disease, there is a need for targeted health education, better sanitation, and easier access to healthcare. These steps can help protect children and control the disease in the long term. The study also recommends longitudinal or mixed-methods research, which will provide broader insight into schistosomiasis.
